# Deep learning identifies morphological features in breast cancer predictive of cancer *ERBB2* status and trastuzumab treatment efficacy

**DOI:** 10.1038/s41598-021-83102-6

**Published:** 2021-02-17

**Authors:** Dmitrii Bychkov, Nina Linder, Aleksei Tiulpin, Hakan Kücükel, Mikael Lundin, Stig Nordling, Harri Sihto, Jorma Isola, Tiina Lehtimäki, Pirkko-Liisa Kellokumpu-Lehtinen, Karl von Smitten, Heikki Joensuu, Johan Lundin

**Affiliations:** 1 Institute for Molecular Medicine Finland (FIMM), Nordic EMBL Partnership for Molecular Medicine, University of Helsinki, Helsinki, Finland; 2 iCAN Digital Precision Cancer Medicine Flagship, Helsinki, Finland; 3 Department of Women’s and Children’s Health, International Maternal and Child Health, Uppsala University, Uppsala, Sweden; 4 Physics and Technology, Research Unit of Medical Imaging, University of Oulu, Oulu, Finland; 5 Department of Diagnostic Radiology, Oulu University Hospital, Oulu, Finland; 6 Ailean Technologies Oy, Oulu, Finland; 7 Department of Pathology, Medicum, University of Helsinki, Helsinki, Finland; 8 Department of Cancer Biology, BioMediTech, University of Tampere, Tampere, Finland; 9 Helsinki University Hospital, Helsinki, Finland; 10 Department of Oncology, Tampere University Hospital, Tampere, Finland; 11 Eira Hospital, Helsinki, Finland; 12 Department of Oncology, Helsinki University Hospital and University of Helsinki, Helsinki, Finland; 13 Department of Global Public Health, Karolinska Institutet, Stockholm, Sweden

**Keywords:** Breast cancer, Tumour biomarkers, Biomarkers

## Abstract

The treatment of patients with *ERBB2* (HER2)-positive breast cancer with anti-*ERBB2* therapy is based on the detection of *ERBB2* gene amplification or protein overexpression. Machine learning (ML) algorithms can predict the amplification of *ERBB2* based on tumor morphological features, but it is not known whether ML-derived features can predict survival and efficacy of anti-*ERBB2* treatment. In this study, we trained a deep learning model with digital images of hematoxylin–eosin (H&E)-stained formalin-fixed primary breast tumor tissue sections, weakly supervised by *ERBB2* gene amplification status. The gene amplification was determined by chromogenic in situ hybridization (CISH). The training data comprised digitized tissue microarray (TMA) samples from 1,047 patients. The correlation between the deep learning–predicted *ERBB2* status, which we call H&E-*ERBB2* score, and distant disease-free survival (DDFS) was investigated on a fully independent test set, which included whole-slide tumor images from 712 patients with trastuzumab treatment status available. The area under the receiver operating characteristic curve (AUC) in predicting gene amplification in the test sets was 0.70 (95% CI, 0.63–0.77) on 354 TMA samples and 0.67 (95% CI, 0.62–0.71) on 712 whole-slide images. Among patients with *ERBB2*-positive cancer treated with trastuzumab, those with a higher than the median morphology–based H&E-*ERBB2* score derived from machine learning had more favorable DDFS than those with a lower score (hazard ratio [HR] 0.37; 95% CI, 0.15–0.93; *P* = 0.034). A high H&E-*ERBB2* score was associated with unfavorable survival in patients with *ERBB2*-negative cancer as determined by CISH. *ERBB2*-associated morphology correlated with the efficacy of adjuvant anti-*ERBB2* treatment and can contribute to treatment-predictive information in breast cancer.

## Introduction

Convolutional neural networks (CNN) are powerful pattern recognizers that have been used successfully to classify cancer tissue morphology and predict patient outcomes^1–7^. In addition, recent studies have suggested that information about gene mutations and other molecular features of cancerous tissue can also be obtained from tissue morphology using only weakly supervised machine learning^2,8^. Examples of CNN-mediated identification of molecular events associated with cancer include the prediction of up to six different gene mutations in lung cancer^9^ and microsatellite instability in colorectal cancer^10^. It is, however, not known whether the CNN-derived morphological features that predict the molecular status of a tumor also could be used to guide the choice of molecularly targeted therapies.

In recent studies on breast cancer, a CNN trained on tissue histomorphology predicted the steroid hormone receptor status, expression of the Ki-67 protein (indicator of cell proliferation), human epidermal growth factor receptor 2 (*ERBB2,* alias *HER2; HGNC:3430*) and a series of other tissue biomarkers in a large proportion of the patients^11,12^. A specific question related to breast cancer is therefore whether a CNN trained to predict the *ERBB2* status of a tumor also could predict the efficacy of anti-*ERBB2* adjuvant treatment.

The identification of amplified *ERBB2* as a major driver in approximately 20% of breast cancers and the subsequent development of anti-*ERBB2*-targeted therapies, such as trastuzumab, turned out to be a major success^13^. Patients with *ERBB2*-positive breast cancer had unfavorable survival rates in the past, but systemic regimens with *ERBB2*-targeted agents have substantially improved survival outcomes^14^. Patients who benefit from *ERBB2*-targeted treatments are usually identified by demonstrating the presence of *ERBB2* amplification with in situ hybridization or an excess of *ERBB2* tyrosine kinase protein with immunohistochemistry. The criteria for defining of *ERBB2*-positive cancer are generally accepted, but they have required modification with time, and there are some borderline cases that are challenging to classify^15^. Therefore, there is a need for more accurate approaches to predict the efficacy of anti-*ERBB2* treatment.

In the current study, we explored whether a CNN weakly supervised by tumor *ERBB2* gene amplification status as determined by chromogenic in situ hybridization (CISH) and trained with standard hematoxylin and eosin (H&E) stained tissues samples, can predict breast cancer *ERBB2* status. We refer to the CNN-based prediction as H&E-*ERBB2* score. Additionally, we explored whether H&E-*ERBB2* score is associated with survival in patient populations treated with or without adjuvant trastuzumab. Interestingly, the CNN trained in the current study was not only a statistically significant and an independent predictor of the *ERBB2* status but also identified patients with CISH *ERBB2*-positive cancer who benefited more from trastuzumab. In addition, the H&E-based *ERBB2* predictor, i.e. H&E-*ERBB2* score identified patients who were CISH *ERBB2* negative but according to the CNN exhibited *ERBB2*-positive cancer-like tissue morphological features and had unfavorable outcomes.

## Methods

### Patient series

The study was based on cancer tissue samples, clinicopathological data, and follow-up data from three independent breast cancer cohorts: the FinProg patient series^16^, the FinProg validation series^17^, and the FinHer clinical trial (ISRCTN76560285)^18^. The FinProg patient series, with data from 2,936 patients, is a nationwide cohort that includes approximately 50% of all women diagnosed with breast cancer in Finland in 1991 or 1992^19^ and covers most (93%) of the patients with breast cancer diagnosed within five selected geographical regions. The FinProg validation series consisted of 565 patients diagnosed mainly in the Helsinki region who were treated at the Departments of Surgery and Oncology, Helsinki University Hospital, from 1987 to 1990. The outcome and cause of death data were retrieved from the files of the Finnish Cancer Registry and Statistics Finland. Breast cancer specific survival was used as an endpoint in the present study. Corresponding clinical information and pathologic tumor characteristics, including histological grade were available from the hospital and laboratory records.

The FinHer trial (ISRCTN76560285) was an open-label multicenter randomized trial that included 1,010 patients in Finland in 2000–2003^20^. Eligible women were ≤ 65 years of age, had undergone breast surgery with axillary nodal dissection, and had either axillary lymph node-positive or high-risk node-negative cancer. Histological grading of cancer was done by pathologists at the time of the diagnosis according to the World Health Organization guidelines. Cancer estrogen receptor (ER), progesterone receptor (PR), and *ERBB2* expression were determined by immunohistochemistry. For all patient samples considered positive for *ERBB2* expression by immunohistochemistry (either 2 + or 3 + on a scale from 0 to 3 +), *ERBB2* amplification status was determined centrally by chromogenic in situ* hybridization* (CISH). Cancers with ≥ 6 gene copies were considered *ERBB2*-positive. The patients were randomly assigned to receive three cycles of docetaxel or vinorelbine, followed in both groups by three cycles of fluorouracil, epirubicin, and cyclophosphamide (FEC). The 232 (23.0%) patients with *ERBB2*-positive cancer underwent a second randomization either to receive concomitant intravenous trastuzumab for 9 weeks or not to receive trastuzumab. Distant disease-free survival (DDFS) was used as the endpoint in the FinHer trial. The median follow-up time was 5.2 years after random assignment^18^.

### Tumor tissue microarray preparation and digitization

Tumor tissue microarrays (TMAs) were prepared from each patient’s representative formalin-fixed paraffin-embedded breast cancer samples^16^. We prepared 23 TMA blocks, each containing 50 to 144 tumor samples, from the 2,306 breast tumor samples available. H&E-stained TMAs (FinProg) and whole-slide tissue sections (FinHer) were digitized using a whole-slide scanner (Pannoramic 250 FLASH, 3DHISTECH Ltd., Budapest, Hungary) (see eMethods 1 in Supplement). The slides were scanned with a 20 × objective lens and the pixel size of the whole-slide images (WSIs) was 0.24 μm. The WSIs were compressed (Enhanced Compressed Wavelet format) and digital stain normalization was performed to adjust for the color intensity variation in the H&E stains^21^.

### Determination of ERBB2 amplification status

In the FinProg cohorts, *ERBB2* amplification was quantified by CISH on TMA cores as described previously^16^. In the FinHer cohort, *ERBB2* amplification was determined by CISH on full tumor sections as part of the FinHer study^18^. Binary *ERBB2* amplification status was derived for each tissue sample^16,18^ from the original CISH images. In this case cell-level information aggregates to the entire tissue sample label, which we refer to as weakly labeled data.

### Training of deep neural networks

**Figure 1:**
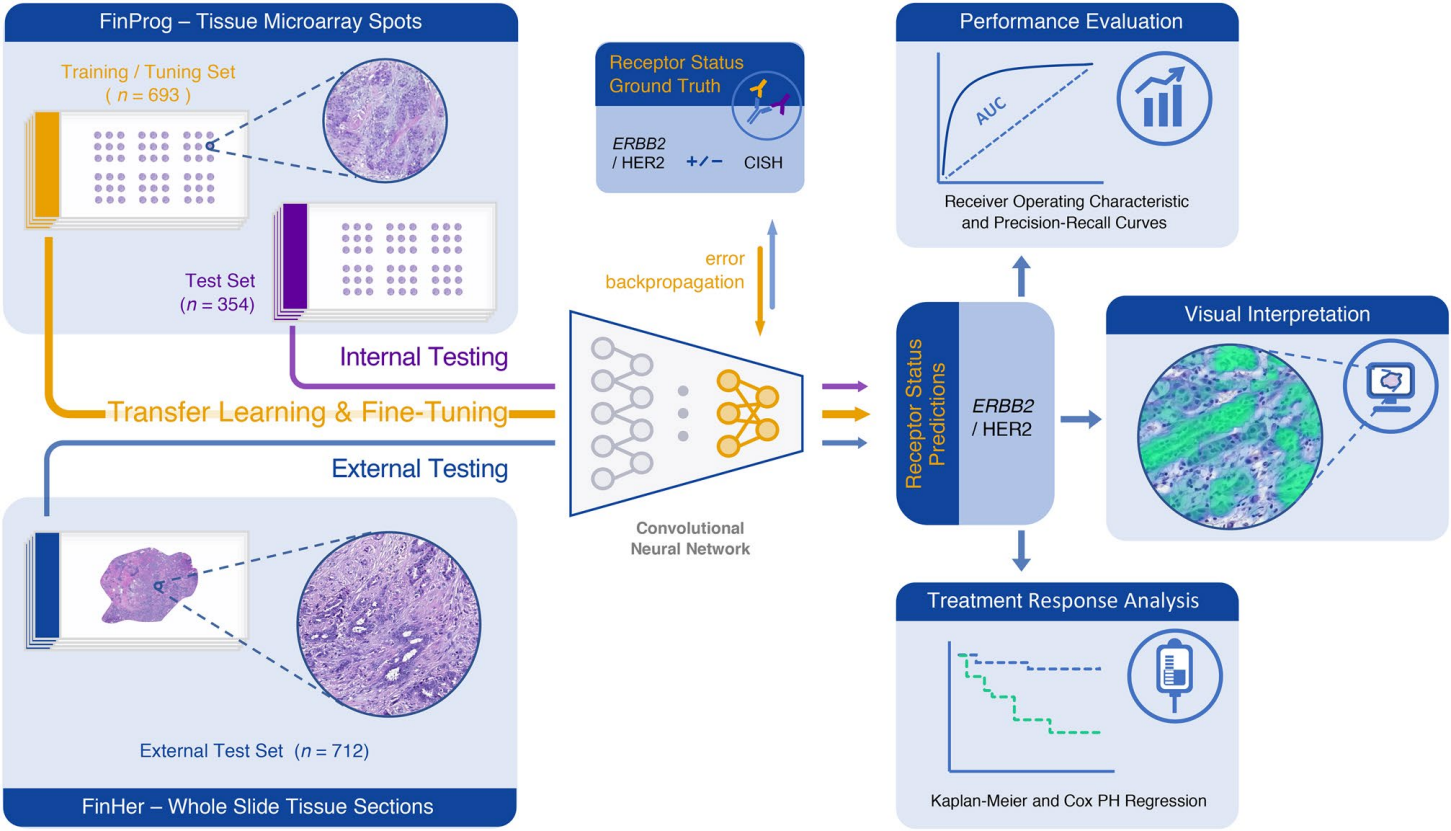
Flow chart of the study design. Deep convolutional neural networks were trained on hematoxylin and eosin stained tissue microarray spots from a nationwide breast cancer series (FinProg) to predict the *ERBB2* gene amplification status of the primary tumor. The networks were trained using a transfer learning approach with ImageNet pretrained weights, and only the deepest layers (colored in yellow) were finetuned by minimizing the focal loss, weakly supervised by the ground truth *ERBB2* gene amplification status as determined by chromogenic in situ hybridization. At the test phase, the networks generate probabilities of *ERBB2* amplification (the H&E-*ERBB2* score). The classification accuracy was summarized with receiver operating characteristic and precision-recall curves. Additionally, we applied Kaplan–Meier plots and Cox regression analysis to correlate the H&E-*ERBB2* scores with patient treatment outcome data.

We built a deep neural network comprised of a stack of convolutional layers taken from a squeeze-and-excitation CNN architecture^22^ (se-resnetxt50_32 × 4d) and a fully connected block. We employed a transfer learning approach by initializing the convolutional layers with the weights trained on ImageNet^23^. The training configuration and CNN parameters can be found in the Supplement. All experiments were carried out using stratified fivefold cross-validation, i.e., preserving target class distributions within each of the folds. Training, validation and testing of the networks was performed on image tiles extracted from the FinProg data. Additional (external) evaluation was performed on FinHer tissue samples. Detailed description of the tile extraction procedures described in the supplementary. Image tiles used for training, originated from the FinProg patient samples that were not used for model testing. To ensure generalization of the models, we additionally tested them on a completely independent patient cohort (FinHer). Data utilization for training and testing is depicted on Fig. 1. Patient splits are summarized in Table 1. Resulting networks predicted probabilities of *ERBB2* amplification that we refer to as H&E-*ERBB2* score. All deep learning models were trained and evaluated using *PyTorch* deep learning library^24^.

**Table 1:** Biological characteristics of breast cancers and patient survival.

	FinProg Patient Series (original and validation)								FinHer patient Series			
	Training and tuning (*N* = 693)		Internal test set (*N* = 354)		Included patients (*N* = 1047)		Total (*N* = 1299)		External test set (*N* = 712)		Total (*N* = 1009)	
Variables:	*n*	%	*n*	%	*n*	%	*n*	%	*n*	%	*n*	%
**Histological grade**												
1	98	*14.1*	68	*19.2*	166	*15.9*	226	*17*	95	*13.3*	150	*14.9*
2	244	*35.2*	127	*35.9*	371	*35.4*	450	*35*	276	*38.8*	397	*39.3*
3	168	*24.2*	68	*19.2*	236	*22.5*	273	*21*	303	*42.6*	414	*41.0*
NA	183	*26.4*	91	*25.7*	274	*26.2*	350	*27*	38	*5.3*	48	*4.8*
CISH *ERBB2*												
Positive	136	*19.6*	66	*18.6*	202	*19.3*	216	*17*	164	*23.0*	233	*23.1*
Negative	557	*80.4*	288	*81.4*	845	*80.7*	944	*73*	548	*77.0*	776	*76.9*
NA							139	*10*				
**ER**												
Positive	472	*68.1*	243	*68.6*	715	*68.3*	812	*63*	501	*70.4*	729	*72.2*
Negative	221	*31.9*	111	*31.4*	332	*31.7*	364	*28*	211	*29.6*	280	*27.8*
NA							123	*9*				
**PR**												
Positive	367	*53.0*	191	*54.0*	558	*53.3*	638	*49*	413	*58.0*	584	*57.9*
Negative	326	*47.0*	163	*46.0*	489	*46.7*	539	*41*	299	*42.0*	425	*42.1*
NA							122	*9*				
Survival*												
Censored	483	*69.7*	254	*71.8*	737	*70.4*	979	*75*	593	*83.3*	846	*83.8*
Uncensored	210	*30.3*	100	*28.2*	310	*29.6*	205	*16*	119	*16.7*	163	*16.2*
NA							115	*9*				

*FinProg—Breast cancer-specific survival; FinHer—Distant disease-free survival (DDFS); NA—not available.

### H&E-ERBB2 score maps and activation maps

The continuous H&E-*ERBB2* score values were overlaid on top of the corresponding locations on the digitized whole-tissue section images to generate H&E-*ERBB2* score maps, i.e. study the distribution of *ERBB2*-associated features. To further identify the subregions in the H&E-stained breast tumor sections that were most informative for a high versus low H&E-*ERBB2* score, we used gradient-weighted class activation mapping i.e. Grad-CAM^25^.

### Statistical analysis

To perform analysis on the FinHer whole-slide sections we first pulled tile-level H&E-*ERBB2* scores to a whole-slide score by taking a median value within each slide. The area under the receiver operating characteristic (ROC) curve (AUC) was used to quantify the capability of the model to distinguish *ERBB2*-positive and *ERBB2*-negative cancers as assessed by CISH. Because of imbalanced data with a larger proportion of CISH *ERBB2*-negative cancers, we also generated precision-recall curves (PRC), which depict the positive predictive value of the classifier (precision) at each test sensitivity (recall) value across various thresholds^26^. Average precision (AP) was calculated from the PRC curves. Confidence intervals were calculated for both AUCs and APs using a stratified bootstrapping technique with 2,000 iterations. To assess whether the H&E-*ERBB2* score was independent of the grade of tumor differentiation, we fitted a logistic regression model with *ERBB2* CISH status as the dependent variable. We used *statsmodels*^27^ python package to perform logistic regression analysis.

To assess the relationship between the prediction of cancer *ERBB2* status and distant disease-free survival (DDFS), we applied Cox regression. DDFS in the FinHer series was computed from the date of randomization to the first detection of metastases outside of the locoregional area or to death from any cause, whichever occurred first. Kaplan–Meier survival curves were drawn using the median value of the predictor as the cut-off.

### Ethical approval

The use of the FinProg patient series of breast cancer samples and the clinical data was approved by the operative Ethics Committee of the hospital district of Helsinki and Uusimaa (94/13/03/02/2012). Also, the National Supervisory Authority for Welfare and Health (Valvira) approved the use of human tissues (7717/06.01.03.01/2015). The National Committee on Medical Research Ethics operates in conjunction with Valvira. Profiling of tumors from the FinHer patient series was approved by the institutional review board of Helsinki University Hospital (HUS 177/13/03/02/2011). FinHer study participants provided written informed consent. All methods were carried out in accordance with relevant guidelines and regulations.

### Consent for publication

Not applicable.

## Results

### Patient selection criteria

We included 1,886 patients from the original FinProg patient series and 427 patients from the FinProg validation series (see Supplementary Fig. 1). The patients that had missing data on follow-up, carcinoma in situ, distant metastases at the time of the diagnosis and those who did not undergo surgery for the primary tumor were excluded. Additionally, we excluded patients with synchronous or metachronous bilateral breast cancer and those with other malignancies. The median follow-up of the patients alive at the end of follow-up was 15.4 years (range, 15.0–20.9 years). Further, for the current study we excluded the patients with missing, detached or not representative tissue spots, e.g. tissue sample area < 0.02 mm^2^, and patients with *ERBB2* data. After all the exclusions (see Supplementary Fig. 1), 1,047 tissue microarray spots (one per patient) were retained for further analysis (Table 1). In the FinProg series 30.3% of the patients (n = 310) died of breast cancer by the end of the follow up. Among the FinHer patients, whole-slide tumor tissue sections from the primary breast cancer were available from 712 (70.6%) patients (Supplementary Fig. 2) and 16.7% of the patients (n = 119) developed distant metastasis by the end of follow-up (i.e. were uncensored in the survival analysis).

### Prediction of tumor ERBB2 status from tissue morphology

**Figure 2:**
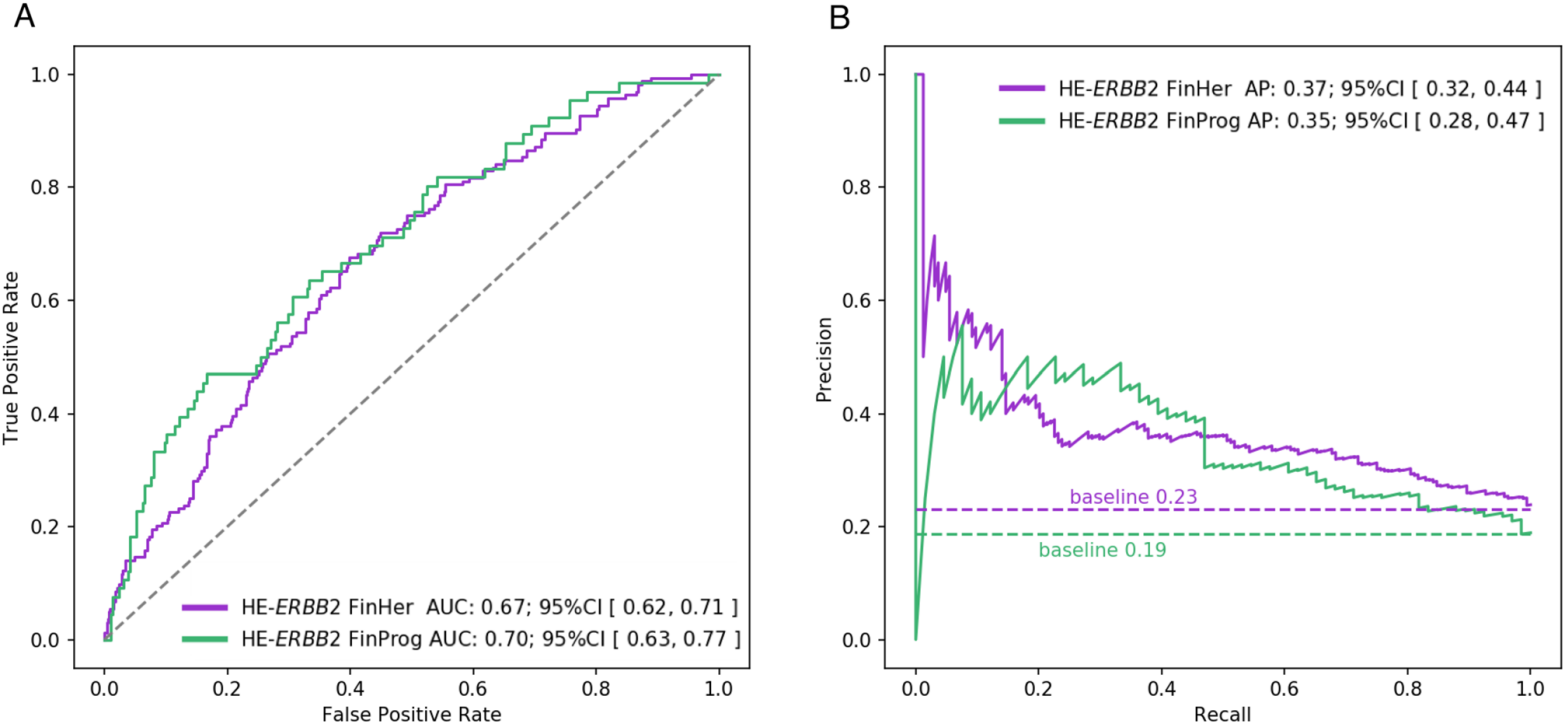
The H&E-*ERBB2* score is a significant predictor of tumor *ERBB2* gene amplification as determined by chromogenic in situ hybridization. The accuracy of neural network-mediated prediction of breast cancer *ERBB2* status was evaluated with (**A**) receiver operating characteristic (ROC) curves and (**B**) precision-recall curves (PRC). The results are presented for both the internal (FinProg) and the external (FinHer) test sets. Area under the ROC curve (AUC) and average precision (AP) were calculated with 90% confidence intervals. The PRC curves were compared to the baseline precision, i.e. precision of a random classifier. The baseline is the ratio of *ERBB2*-positive samples over the total number of *ERBB2*-positive and *ERBB2*-negative samples as determined by chromogenic in situ hybridization.

We first trained a CNN to predict breast cancer *ERBB2* status on 693 H&E-stained patient samples from the FinProg series. To validate the generalization of the trained models, we evaluated them on two test sets. First, we assessed the performance of the models on a FinProg held-out tissue microarray samples (n = 354). Then, the models were tested on a completely independent set of 712 whole-slide tissue sections from the FinHer patient series (Table 1). The overall setup of the computational experiment and analysis is depicted in Fig. 1. On a randomly selected internal test set from the FinProg series, the model achieved an AUC of 0.70 (95% confidence interval (CI), 0.63–0.77) and an AP of 0.35 (95% CI, 0.28–0.47) with a baseline AP of 0.19. The accuracy of individual models trained within fivefold cross-validation is reported on the Supplementary Fig. 3. Additionally, contingency table calculated on the FinProg hold out data is depicted in Supplementary Fig. 4. When we next applied the model on 712 breast cancer whole-slide tissue images from the FinHer dataset (external test set), the CNN predicted *ERBB2* status with an AUC of 0.67 (95% CI, 0.62–0.71) and an AP of 0.37 (95% CI, 0.32–0.44) with a baseline AP of 0.23 (Fig. 2). These results suggest that the H&E-*ERBB2* score is a predictor of *ERBB2* status as determined by CISH and that the model generalizes from small tumor areas (tissue microarray spots) to whole-slide samples from an independent test cohort.

### H&E-ERBB2 score independent of tumor histological grade

Tumor histological grade also predicted cancer *ERBB2* status in the FinProg series (*P* < 0.001). When both the H&E-*ERBB2* score and histological grade were included in a logistic regression analysis as covariates, the H&E-*ERBB2* score remained as an independent predictor of breast cancer *ERBB2* status in the FinProg test set (*P* = 0.005) and also in the FinHer external test set (*P* < 0.001). Additional details of the logistic regression analysis are provided in supplementary Tables 1 and 2 respectively. This suggests that the deep learning model identified morphological patterns associated with *ERBB2* gene amplification not explained by grade of differentiation.

### ERBB2-associated morphology predicts distant disease-free survival

**Figure 3:**
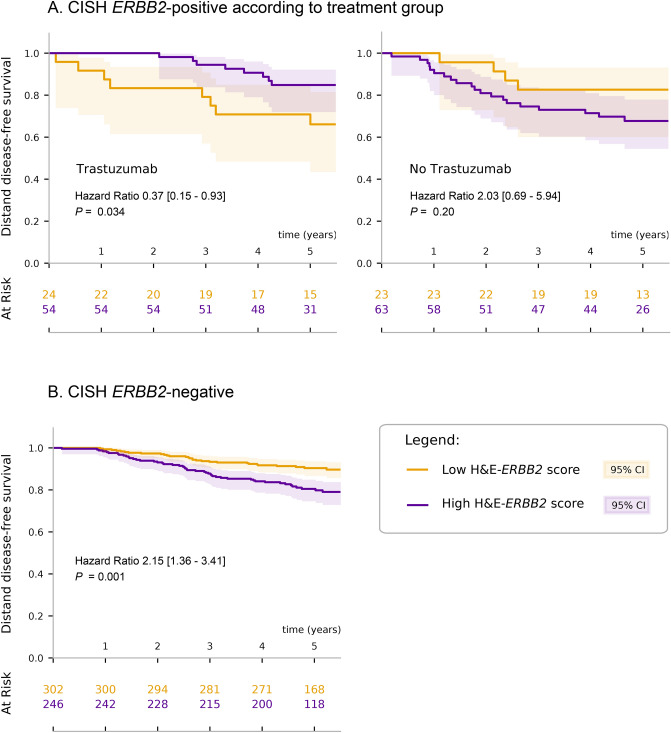
Cancer tissue morphology-based H&E-*ERBB2* score and distant disease-free survival. (**A**) Evaluation of H&E-*ERBB2* scores and distant disease-free survival (DDFS) in patients with *ERBB2*-positive breast cancer as determined by chromogenic in situ hybridization (CISH) in the FinHer trial series. Left panel: Patients treated after breast surgery with chemotherapy plus adjuvant trastuzumab. Right panel: Patients treated with chemotherapy but without trastuzumab. (**B**) The DDFS of CISH *ERBB2*-negative patients in the FinHer series stratified by the H&E-*ERBB2* score. None of the patients judged *ERBB2*-negative by CISH received adjuvant trastuzumab.

As CISH *ERBB2*-positive patients were randomly assigned to receive or not receive adjuvant trastuzumab as part of the FinHer trial, we performed the analysis in each of the treatment groups separately. CISH *ERBB2*-positive patients with high H&E-*ERBB2* score and treated with trastuzumab had a more favorable DDFS than those who had a low H&E-*ERBB2* score (HR, 0.37; 95% CI, 0.15–0.93; *P* = 0.034). CISH *ERBB2*-positive patients not treated with trastuzumab and who had a high H&E-*ERBB2* score tended to have a less favorable DDFS (HR, 2.03; 95% CI, 0.69–5.94; *P* = 0.20; Fig. 3A). This suggests that the H&E-*ERBB2* score could contribute to a more accurate prediction of trastuzumab efficacy than the CISH *ERBB2* status alone.

Among the 548 patients who were *ERBB2*-negative by CISH and who, therefore, were not eligible to receive trastuzumab in the FinHer trial, 246 (45%) had a high H&E-*ERBB2* score and 302 (55%) had a low score, as determined by a median H&E-*ERBB2* score on the entire external test set. In this subset, the patients with a high H&E-*ERBB2* score had a less favorable survival than the patients with a low score (HR 2.15; 95% CI, 1.36–3.41; *P* = 0.001; Fig. 3B). All together, these observations suggest that some of the CISH *ERBB2*-negative patients might potentially benefit from anti-*ERBB2* treatment and those can be detected via analyzing conventional H&E-stained cancer tissue samples.

### Activation maps for the deep learning model trained to predict the ERBB2 gene amplification status

**Figure 4:**
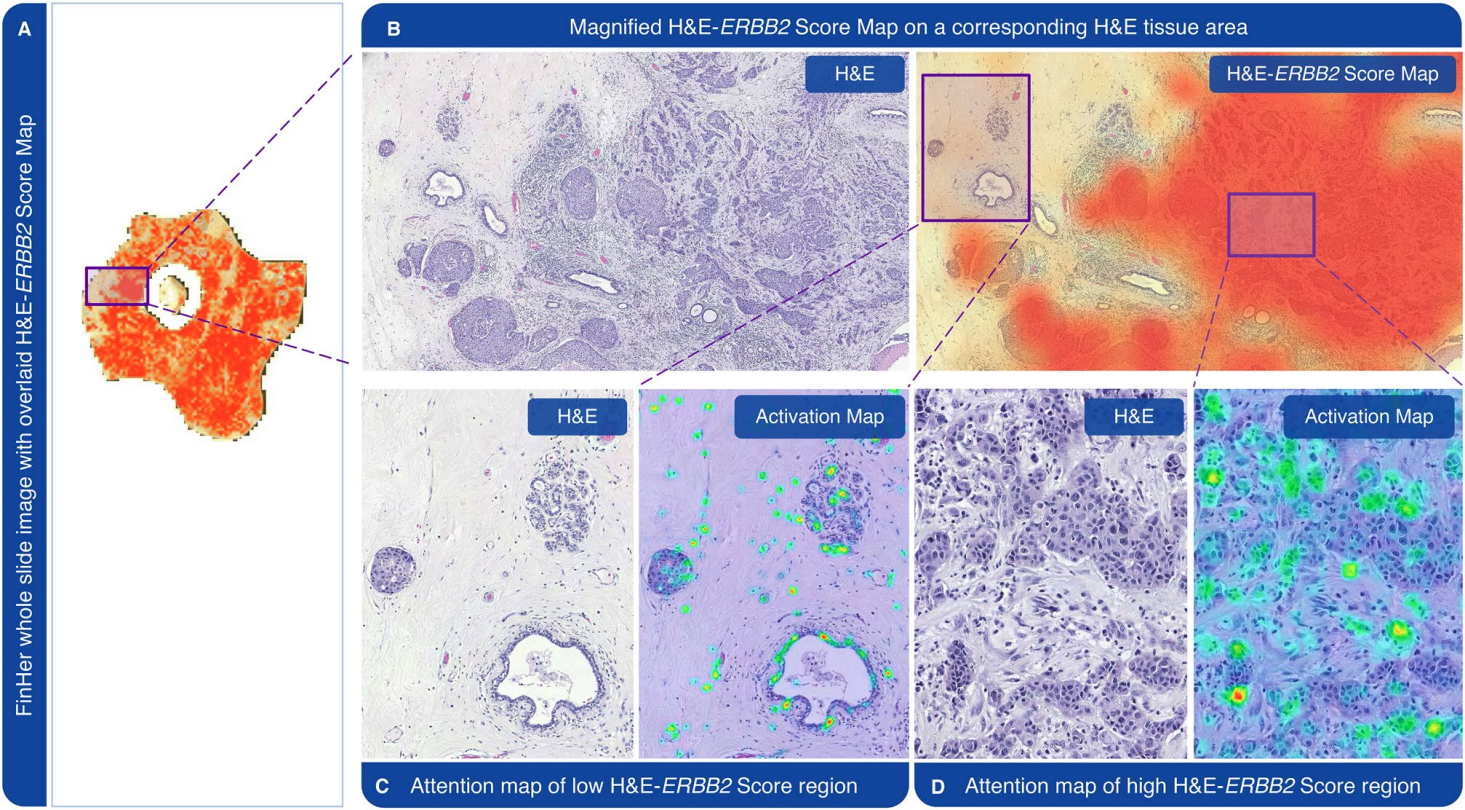
H&E-*ERBB2* score and Grad-CAM activation maps. (**A**) H&E-*ERBB2* Score Map as predicted by the convolutional neural network (CNN) based on a hematoxylin eosin (H&E) stained sample from the FinHer cohort. The Score Map was overlaid as a heatmap on top of the H&E-stained whole-slide tissue image, representing variable levels of the CNN-derived H&E-*ERBB2* score. (**B**) Left: Magnified image of the box shown in panel A. Right: Areas representing high H&E-*ERBB2* Scores indicated with red. (**C**) Grad-CAM activation map of a region predicted to have a low H&E-*ERBB2* score. (**D**) Grad-CAM activation map of a region with a high H&E-*ERBB2* score. The high score areas indicate clusters of cancerous epithelial cells. The sample presented is *ERBB2*-positive by chromogenic in situ hybridization and had a high overall H&E-*ERBB2* score.

We observed substantial variability of the H&E-*ERBB2* score within and across the samples, suggesting that the *ERBB2* associated patterns learned by the CNN are heterogeneously distributed in the tissue (Fig. 4). According to the Grad-CAM activation maps, the tissue morphological features that were most predictive of *ERBB2* gene amplification based on the CNN analysis were regions of tumor epithelium and in situ carcinoma components, as well as individual epithelial cells and fibroblasts in the stromal regions (Fig. 4C,D).

## Discussion

In this study, we have shown that a CNN trained on a primary breast tumor tissue morphology is able to learn patterns predictive of breast cancer *ERBB2* gene amplification status as assessed by chromogenic in situ hybridization. More importantly, our findings generalized from the limited tumor areas, i.e. tissue microarray (TMA) spots to whole slide tumor sections and samples from multiple centers. This suggests that *ERBB2* status is reflected in breast cancer tissue architecture, and that the information can be captured by and quantified with machine learning.

Further, we have shown that *ERBB2* amplification–associated morphology reflected by a high H&E-*ERBB2* score is correlated with the efficacy of adjuvant trastuzumab therapy and predicts a significantly more favorable distant disease-free survival in CISH *ERBB2*-positive patients. Conversely, in CISH *ERBB2* positive patients who had a high H&E-*ERBB2* score but were randomized not to receive trastuzumab, a trend towards a less favorable DDFS was seen. Similarly, in CISH *ERBB2*-negative patients who were not eligible to receive trastuzumab, a high H&E-*ERBB2* score was associated with a significantly less favorable DDFS. This indicates that the *ERBB2*-associated morphology may contain significant therapy-predictive information to complement the molecular (CISH-based) analysis.

Our findings related to morphology-based prediction of the *ERBB2* status are in line with those from a recent study, in which a deep learning-based method was used to predict *ERBB2* and a series of other biomarkers in breast cancer from H&E-stained tissue microarray spots^11,12^. In our work, the accuracy of the prediction of the *ERBB2* status was on a similar level (AUC 0.70) as in the previous study by Shamai et al. (AUC 0.74)^11^ when validated on tissue microarray samples. Our hold-out TMA test set was smaller than theirs, which could explain a lower accuracy. Shamai et al. showed that increasing the number of samples for training from 1,000 tumor samples to 4,000 correspondingly increased the AUC from 0.66 to 0.74^11^ suggesting that our results may be considered on par with theirs taking into account the number of training samples.

In this study, we were able to address several of the limitations raised in the previous study such as expanding the testing material from small tumor areas (TMAs) to whole slide samples and from a single center to a nationwide setting and multiple centers.

To better understand what *ERBB2*-associated morphological patterns the CNN has learned, we visualized network scores and activation maps on top of the corresponding histological images. In general, activation of the CNN-based *ERBB2* predictor was focused on tumor epithelium, and larger and smaller nests of malignant epithelial cells, rather than stromal areas. In certain cases, the activation of the CNN was focused on individual cancerous epithelial cells. This is in line with what would be expected, given that the *ERBB2* gene amplification and protein overexpression is occurring in cancerous epithelial cells.

Related to the predictive information of the H&E-*ERBB2* score that we found to complement the *ERBB2* gene amplification status, one could speculate that the CNN has learned auxiliary tumor features associated with efficacy of trastuzumab therapy and DDFS. While features learned with weakly supervised machine learning may not necessarily represent the actual target (e.g. cells with a specific mutation or gene amplification), the tissue patterns identified using this approach can still yield relevant information about tumor biology^2^. Prognostic tissue microarchitectural features in breast cancer have been previously described and captured both with machine learning^1^ and conventional image analysis^28^. In these studies, a series of features including the tumor-stroma interface, distance between stromal regions and size of tumor nests were reported to be associated with survival in breast cancer^1,28^. In that context, the relationship between the H&E-*ERBB2* score and molecular subtypes, TILs, and other prognostic tissue biomarkers should be further explored.

Future studies should aim to explore these tissue microarchitectural features in more detail, for example with multiplexed methods that allow morphological and molecular characterization (e.g. multiplexed immunohistochemistry or in situ sequencing) of the same tissue sections or using cell-level registration of consecutive tissue sections.

Our study has certain limitations that should be addressed in future studies. We did not have access to consecutive sections of samples with *ERBB2* gene amplification results as determined by chromogenic in situ hybridization (CISH) or *ERBB2* protein expression assessed with immunohistochemistry (IHC). Consecutive slides or multiplexed analysis of the same tissue section with H&E, CISH, IHC or in situ sequencing could allow improved localization of the *ERBB2*-associated features in the H&E morphology that predicted the efficacy of anti-*ERBB2* treatment in our study. Overall accuracy and especially the specificity of the *ERBB2* predictions could be improved by for example incorporating more training samples from external datasets. *ERBB2* expression heterogeneity and its effect on survival should be also analyzed on the whole-slide sections. Another limitation was the restricted number of patients in some of the subgroups. To confirm our findings, analyses of additional patient series treated with adjuvant or neoadjuvant anti-*ERBB2*-targeted therapy are needed.

To the best of our knowledge, the present study is the first to show that a deep learning algorithm trained on tissue morphology and weakly supervised by the molecular status can learn patterns that predict the efficacy of adjuvant systemic therapy in patients with breast cancer. The present study suggests that machine learning can be used to extract predictive information when applied to routine tumor tissue sections and may help in identifying patients with *ERBB2*-positive cancer who would benefit the most from adjuvant trastuzumab. Our findings also suggest that some patients whose cancer is identified as *ERBB2*-negative by CISH do in fact have *ERBB2*-associated morphology, and thus potentially could benefit from targeted anti-*ERBB2* therapy. This warrants further studies, perhaps with the H&E-*ERBB2* score as a companion diagnostic assay in clinical trials with agents that show clinical activity in *ERBB2*-low-expression breast cancer^29^. Further research should also elucidate the amount of clinically relevant and actionable information that remains to be extracted from ubiquitous, inexpensive H&E-stained samples, such as features that predict the efficacy of hormone therapy and other molecularly targeted treatments.

## Supplementary Information

Supplementary Information.

## Data Availability

The data that support the findings of this study were used under a license for the current study, and some restrictions apply to their availability. The data are available from the authors upon reasonable request and with permission from the University of Helsinki.

## References

[1] Beck AH (2011). Systematic analysis of breast cancer morphology uncovers stromal features associated with survival. Sci. Transl. Med..

[2] Campanella G (2019). Clinical-grade computational pathology using weakly supervised deep learning on whole slide images. Nat. Med..

[3] Ström P (2020). Artificial intelligence for diagnosis and grading of prostate cancer in biopsies: a population-based, diagnostic study. Lancet Oncol..

[4] Skrede O-J (2020). Deep learning for prediction of colorectal cancer outcome: a discovery and validation study. Lancet.

[5] Mobadersany P (2018). Predicting cancer outcomes from histology and genomics using convolutional networks. Proc. Natl. Acad. Sci..

[6] Bychkov D (2018). Deep learning based tissue analysis predicts outcome in colorectal cancer. Sci. Rep..

[7] Courtiol P (2019). Deep learning-based classification of mesothelioma improves prediction of patient outcome. Nat. Med..

[8] Rony, J. *et al.* Deep weakly-supervised learning methods for classification and localization in histology images: a survey. arXiv e-prints arXiv:1909.03354 (2019).

[9] Coudray N (2018). Classification and mutation prediction from non-small cell lung cancer histopathology images using deep learning. Nat. Med..

[10] Kather JN (2019). Deep learning can predict microsatellite instability directly from histology in gastrointestinal cancer. Nat. Med..

[11] Shamai G (2019). Artificial intelligence algorithms to assess hormonal status from tissue microarrays in patients with breast cancer. JAMA Netw..

[12] Rawat RR (2020). Deep learned tissue “fingerprints” classify breast cancers by ER/PR/Her2 status from H&E images. Sci. Rep..

[13] Hayes DF (2019). HER2 and breast cancer—a phenomenal success story. N. Engl. J. Med..

[14] Wilson FR (2018). Herceptin (trastuzumab) in HER2-positive early breast cancer: a systematic review and cumulative network meta-analysis. Syst. Rev..

[15] Wolff AC (2018). Human epidermal growth factor receptor 2 testing in breast cancer: american society of clinical oncology/college of american pathologists clinical practice guideline focused update. J. Clin. Oncol..

[16] Joensuu H (2003). Amplification of erbB2 and erbB2 expression are superior to estrogen receptor status as risk factors for distant recurrence in pT1N0M0 breast cancer. Clin. Cancer Res..

[17] Lundin J, Lundin M, Isola J, Joensuu H (2003). A web-based system for individualised survival estimation in breast cancer. BMJ.

[18] Joensuu H (2006). Adjuvant docetaxel or vinorelbine with or without trastuzumab for breast cancer. N. Engl. J. Med..

[19] Joensuu H (2004). Risk for distant recurrence of breast cancer detected by mammography screening or other methods. JAMA.

[20] Joensuu H (2009). Fluorouracil, epirubicin, and cyclophosphamide with either docetaxel or vinorelbine, with or without trastuzumab, as adjuvant treatments of breast cancer: final results of the FinHer trial. J. Clin. Oncol..

[21] Macenko, M. *et al.* A Method for normalizing histology slides for quantitative analysis. In *Proceedings of the Sixth IEEE International Conference on Symposium on Biomedical Imaging: From Nano to Macro*, 1107–1110 (IEEE Press, 2009).

[22] Hu, J., Shen, L. & Sun, G. Squeeze-and-excitation networks. In *Proceedings of the IEEE Computer Society Conference on Computer Vision and Pattern Recognition*, 7132–7141 (2018).

[23] Russakovsky O (2015). ImageNet large scale visual recognition challenge. Int. J. Comput. Vis..

[24] Paszke A, Wallach H (2019). PyTorch: An Imperative Style, High-Performance Deep Learning Library. Advances in Neural Information Processing Systems.

[25] Selvaraju RR (2020). Grad-CAM: visual explanations from deep networks via gradient-based localization. Int. J. Comput. Vis..

[26] Saito T, Rehmsmeier M (2015). The precision-recall plot is more informative than the roc plot when evaluating binary classifiers on imbalanced datasets. PLoS ONE.

[27] Seabold, S. & Perktold, J. statsmodels: econometric and statistical modeling with python. In *9th Python in Science Conference* (2010).

[28] Roxanis I, Colling R, Kartsonaki C, Green AR, Rakha EA (2018). The significance of tumour microarchitectural features in breast cancer prognosis: a digital image analysis. Breast Cancer Res..

[29] Modi S (2020). Antitumor activity and safety of trastuzumab deruxtecan in rptients with her2-low–expressing advanced breast cancer: results from a phase Ib study. J. Clin. Oncol..

