# Supplementary material for: Deep learning identifies morphological features in breast cancer predictive of cancer ERBB2 status and trastuzumab treatment efficacy
Source: Sci Rep. 2021 Feb 17;11:4037. doi: 10.1038/s41598-021-83102-6 (PMC7890057; doi:10.1038/s41598-021-83102-6)
Supplement: Supplementary file 1 — Supplementary Information. [file 41598_2021_83102_MOESM1_ESM.docx]

# Deep Learning Identifies Morphological Features in Breast Cancer Predictive of Cancer *ERBB2* Status and Trastuzumab Treatment Efficacy

Dmitrii Bychkov^1,2*^, MSc; Nina Linder^1,2,3^, MD, PhD; Aleksei Tiulpin^4,5,6^, PhD; Hakan Kücükel^1,2^, BBA; Mikael Lundin^1^, MD; Stig Nordling^7^, MD, PhD; Harri Sihto^7^, PhD; Jorma Isola^8^, MD, PhD; Tiina Lehtimäki^9^, MD, PhD; Pirkko-Liisa Kellokumpu-Lehtinen^10^, MD, PhD; Karl von Smitten^11^, MD, PhD; Heikki Joensuu^2,12^, MD, PhD; Johan Lundin^1,2,13^, MD, PhD

1 Institute for Molecular Medicine Finland (FIMM), University of Helsinki, Finland;

2 iCAN Digital Precision Cancer Medicine Flagship;

3 Department of Women's and Children's Health, International Maternal and Child Health, Uppsala University, Sweden;

4 Research Unit of Medical Imaging, Physics and Technology, University of Oulu, Oulu, Finland;

5 Department of Diagnostic Radiology, Oulu University Hospital, Oulu, Finland;

6 Ailean Technologies Oy, Oulu Finland;

7 Department of Pathology, Medicum, University of Helsinki, Finland;

8 Department of Cancer Biology, BioMediTech, University of Tampere, Tampere, Finland;

9 Helsinki University Hospital, Helsinki, Finland;

10 Department of Oncology, Tampere University Hospital, Finland;

11 Eira Hospital, Helsinki, Finland;

12 Department of Oncology, Helsinki University Hospital and University of Helsinki, Helsinki, Finland;

13 Department of Global Public Health, Karolinska Institutet, Stockholm, Sweden.

*Corresponding author: Dmitrii Bychkov

# Supplementary

### Description of the Deep Learning Model and Parameters

The features from the last convolutional layer of the neural network (se-resnetxt50_32x4d^1^) were first globally average-pooled and then passed to the fully connected block with a hidden layer of size 16 and a single output neuron. The fully connected block was initialized with random weights. We trained the networks for 49 epochs: during the first three epochs, only the fully connected block was trained; and starting from the fourth epoch, the last two convolutional layers were released and fine-tuned together with the fully connected layers. This strategy prevents the corruption of pretrained convolutional layers at the initial stages of training when the gradient of the fully connected layer is noisy due to its random initialization. All our models were trained by minimizing a focal loss^2^ (*alpha*=0.25, *gamma*=2) using Adam optimizer^3^. We used an initial learning rate of 1*e* –5 and dropped it to 1*e* –6 at epoch 9. Weight decay of 1*e* –4 was added to the loss function. Additionally, during training, we introduced a dropout layer (*P* = 0.4) before the fully connected block.

### Image Data Preparation and Pre-processing

The FinProg TMA spot images were manually quality checked and non-representative, samples with no tumor tissue were excluded. No additional filters were applied to the original FinProg TMA images. During training on FinProg images, we extracted square crops at a random location from the TMA spots. One crop of size 950 x 950 pixels per TMA spot was extracted at each epoch. Thus, at each epoch, the networks were supplied with a different set of crops that originated from various locations of the TMA spots included in the training set. A batch of 16 random crops constituted one training iteration, which corresponded to input tensors of size [950, 950, 3, 16] (height, width, color channels, batch size). Original size of each TMA spot was 3500 x 3500 pixels on average. All input tensors were normalized with mean and standard deviation, as estimated on the training data: mean - [0.8198558, 0.78990823, 0.91205645], std - [0.1421396, 0.15343277, 0.07634846] for RGB channels accordingly. After image normalization, we performed on-the-fly training image augmentations using SOLT data augmentation library (<https://github.com/MIPT-Oulu/solt>) with the following parameters:

- random scaling with 0.5 probability and 0.3 scale range
- random rotation with 0.5 probability and ± 90-degree range
- random shear with 0.5 probability and 0.2 shear range
- random gamma correction with 0.5 probability and 0.3 gamma range

For internal testing on 354 FinProg TMA spots we used a center crop of size 2100 x 2100 pixels and applied no image augmentation.

For evaluations on the external test set – the FinHer series, we extracted non-overlapping (step size 950 pixels) tiles of 950 x 950 pixels from each of the whole slide tissue images. We then applied HistoQC quality control tool^4^ to eliminated tiles that contain artefacts such as out of focus regions, tissue folding etc. Each tile that passed the quality check was processed by five models trained in cross-validation on the FinProg training set. The predictions were averaged to obtain a single tile-level H&E-*ERBB2* score. No test-time image augmentation was performed on the FinHer tiles.

### Image Acquisition

Five-micrometer thick sections were cut from the TMA blocks, stained with hematoxylin and eosin, and digitized with a whole-slide scanner (Pannoramic 250 FLASH, 3DHISTECH Ltd., Budapest, Hungary) equipped with a 20 × objective (numerical aperture 0.80) and a 1 × adapter, and a progressive scan color camera with three separate charge-coupled devices with 1 618 × 1 236 pixels sized 4.40 μm × 4.40 μm (CIS_VCC_F52U25CL, CIS Corporation, Tokyo, Japan). This resulted in an image where one pixel represents an area of 0.22 μm × 0.22 μm. The images were stored in a whole-slide image format (MRX, 3DHISTECH Ltd., Budapest, Hungary), and were further compressed to a wavelet file format (Enhanced Compressed Wavelet, ECW, ER Mapper, Intergraph, Atlanta, GA) with a compression ratio of 1:10. The compressed virtual slides were uploaded to a whole-slide image management server (WebMicroscope, Aiforia Technologies Oy, Helsinki, Finland), where the individual images of the TMA spots were segmented from the whole-slide TMA image, and downloaded as uncompressed portable network graphics (PNG) files.

### Figure 1 - FinProg CONSORT Diagram

FinProg series

(N=2,936)

FinProg series patients available after exclusions (N=1,886)

FinProg Validation series

(N=565)

1,014 did not have tumor samples available, sample was not representative, or tissue spot detached

Combined FinProg

and FinProg Validation set

with tumor tissue available

(N=1,299)

*Exclusions: patients with missing data on follow-up, lobular or ductal carcinoma in situ, synchronous or metachronous bilateral breast cancer or other malignancy (except for basal cell carcinoma or cervical carcinoma in situ), distant metastasis, or who did not undergo surgery of the primary tumor were excluded.

Random split

Internal validation

(N=354)

Training / tuning set

(N=693)

FinProg Validation series patients available after exclusions (N=427)

21 had poor quality tissue microarray spot images

231 had missing gene/receptor status data

Available for algorithm development

(N=1,047)

Combined FinProg

and FinProg Validation set

(N=2,313)

138 excluded*

1,050 excluded*

### Figure 2 - FinHer CONSORT Diagram

Initial cohort

(N=1,009)

289 digital slides were not available

8 slide images were blurred or corrupted

Available for analysis

(N=712)

Figure 3


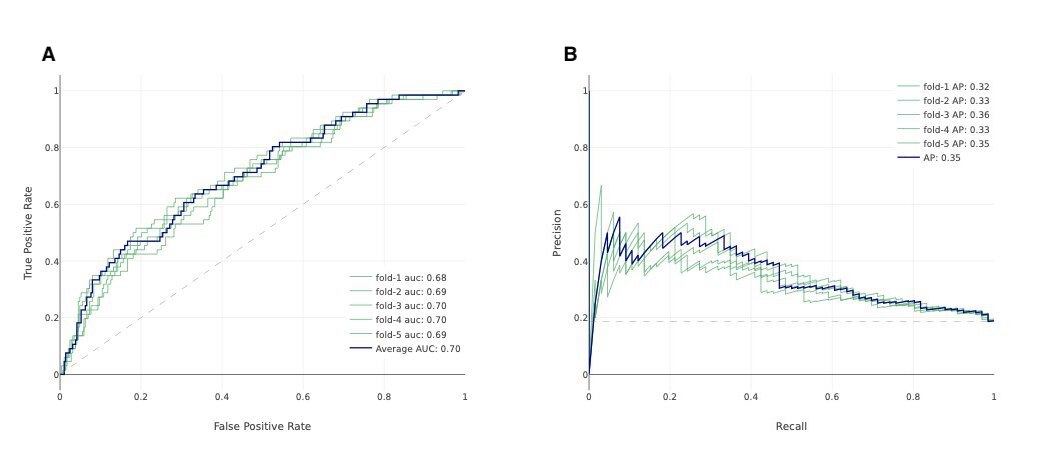


The accuracy of breast cancer *ERBB2* status predictions by individual models trained in 5-fold cross-validation on the FinProg data. Area under the ROC curve (A) and Average Precision (B) as evaluated on the FinProg hold-out data.

Figure 4


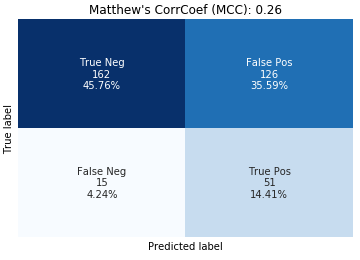


Confusion matrix for the *ERBB2* status predictions on 354 FinProg hold out samples.

Table 1

Output of multivariate logistic regression analysis on the FinProg hold-out set. The results indicate that both H&E-*ERBB2* score and histological grade were independent predictors of CISH ERBB2 status. Number of observations in the hold-out set reduced from 354 to 263 because histological grade for 91 patients was not available.

Table 2

Output of multivariate logistic regression analysis on the FinHer external test set patient samples. The results indicate that both H&E-*ERBB2* score and histological grade were independent predictors of CISH ERBB2 status. Number of observations in the FinHer set reduced from 712 to 694 because histological grade for 18 patients was not available.

REFERENCES

1. Hu, J., Shen, L. & Sun, G. Squeeze-and-excitation networks. in *2018 IEEE/CVF Conference on Computer Vision and Pattern Recognition* 7132–7141 (2018). doi:10.1109/CVPR.2018.00745

2. Lin, T.-Y., Goyal, P., Girshick, R. B., He, K. & Dollár, P. Focal Loss for Dense Object Detection. *2017 IEEE Int. Conf. Comput. Vis.* 2999–3007 (2017).

3. Kingma, D. P. & Ba, J. Adam: a method for stochastic optimization. (2014).

4. Janowczyk, A., Zuo, R., Gilmore, H., Feldman, M. & Madabhushi, A. HistoQC: An Open-Source Quality Control Tool for Digital Pathology Slides. *JCO Clin. Cancer Informatics* 1–7 (2019). doi:10.1200/CCI.18.00157
